# Synchronous Clear Cell and Papillary Renal Cell Carcinoma Collision Tumor Confirmed by Component-Specific Next-Generation Sequencing: A Case Report

**DOI:** 10.7759/cureus.113743

**Published:** 2026-07-31

**Authors:** Yan Li, Lina X Hu

**Affiliations:** 1 Pathology and Translational Pathobiology, Louisiana State University Health Sciences Center Shreveport, Shreveport, USA

**Keywords:** clear cell renal cell carcinoma, collision tumor, next-generation sequencing, papillary renal cell carcinoma, pbrm1, pembrolizumab, rheb, tert, tgfbr1, tumor mutational burden

## Abstract

Synchronous clear cell renal cell carcinoma (ccRCC) and papillary renal cell carcinoma (pRCC) within the same kidney are uncommon, and distinguishing a true collision tumor from a composite neoplasm with shared clonal origin requires molecular confirmation. We report an 83-year-old woman who underwent radical nephrectomy for an incidentally detected left renal mass. Pathologic examination revealed two well-demarcated adjacent tumors (3.3 cm and 3.2 cm) with characteristic morphologic and immunophenotypic features of ccRCC and pRCC, both staged pT1a. Separate next-generation sequencing analyses demonstrated completely distinct molecular profiles: the pRCC harbored RHEB p.Y35C and TERT promoter mutations with TMB-High status, whereas the ccRCC contained truncating alterations in PBRM1, ARID1A, and TGFBR1. No shared somatic mutations were identified, confirming independent clonal origins and establishing this as a true collision tumor. This case illustrates the value of per-component molecular profiling for histologically discordant renal tumors.

## Introduction

Renal cell carcinoma (RCC) comprises a heterogeneous group of malignancies with distinct morphologic, molecular, and clinical characteristics. Clear cell RCC (ccRCC) is the most common subtype, accounting for approximately 75-80% of cases, whereas papillary RCC (pRCC) represents 10-15% and differs substantially in its genomic alterations, biological behavior, and therapeutic modalities [1-3]. A recent population-based cohort study of the Finnish Cancer Registry (1995-2017, n=14,413) confirmed that ccRCC remains the predominant RCC subtype, accounting for 75.5% of cases, compared with only 5.8% for pRCC [4]. This relative rarity of pRCC underscores the growing clinical importance of accurate renal tumor subtyping, particularly when two histologically distinct subtypes are found to coexist within the same kidney. Although multifocal RCC is well recognized, synchronous tumors of different histologic subtypes arising within the same kidney are uncommon [5,6]. When morphologically distinct subtypes coexist, the central question is whether they represent two independent primary tumors (collision tumors) or divergent differentiation within a single neoplasm (composite tumor), a distinction with implications for tumor classification, molecular interpretation, and therapeutic strategy [5,7]. This distinction is not merely academic: whether a tumor is staged and reported as two synchronous primaries or as a single neoplasm with divergent morphology affects prognostic assessment, eligibility for subtype-specific systemic therapies, and whether biomarkers validated in one histologic subtype can be extrapolated to the other.

Most prior reports of synchronous ccRCC and pRCC have relied on morphology and immunohistochemistry without molecular confirmation of clonal independence [5,6,8,9]. The present case differs from these earlier reports in that clonal independence is established directly, through independent, component-specific next-generation sequencing spanning both DNA and RNA platforms, rather than inferred from histologic and immunophenotypic differences alone [8,9]. Comprehensive next-generation sequencing (NGS) can directly assess clonal relationships by comparing the molecular profiles of individual tumor components [1,10]. We present a case of synchronous ccRCC and pRCC confirmed as a true collision tumor by independent DNA and RNA sequencing, following the CARE reporting guidelines. To our knowledge, this is among the first reported cases of a synchronous ccRCC-pRCC collision tumor in which clonal independence was confirmed using combined DNA and RNA next-generation sequencing performed separately on each component.

## Case presentation

An 83-year-old woman was referred to urology after a 6.3 cm left exophytic renal mass with extensive heterogeneous enhancement was identified incidentally on computed tomography (CT) imaging. Her medical history included coronary artery disease, insulin-dependent type 2 diabetes mellitus, hypertension, and hyperlipidemia.

She underwent left open radical nephrectomy and was discharged on postoperative day 4. She returned with a wound separation at the inferior incision and postoperative ileus; both resolved with conservative management, and she was doing well at one-week follow-up. At six weeks and three months postoperatively, nephrology evaluation confirmed stage IIIB chronic kidney disease in the setting of a solitary right kidney; she was continued on renin-angiotensin-aldosterone system inhibition and a sodium-glucose cotransporter-2 inhibitor for renoprotection. No adjuvant oncologic therapy was initiated, and she remained without evidence of disease at surveillance follow-up with urology and oncology.

**Figure 1:**
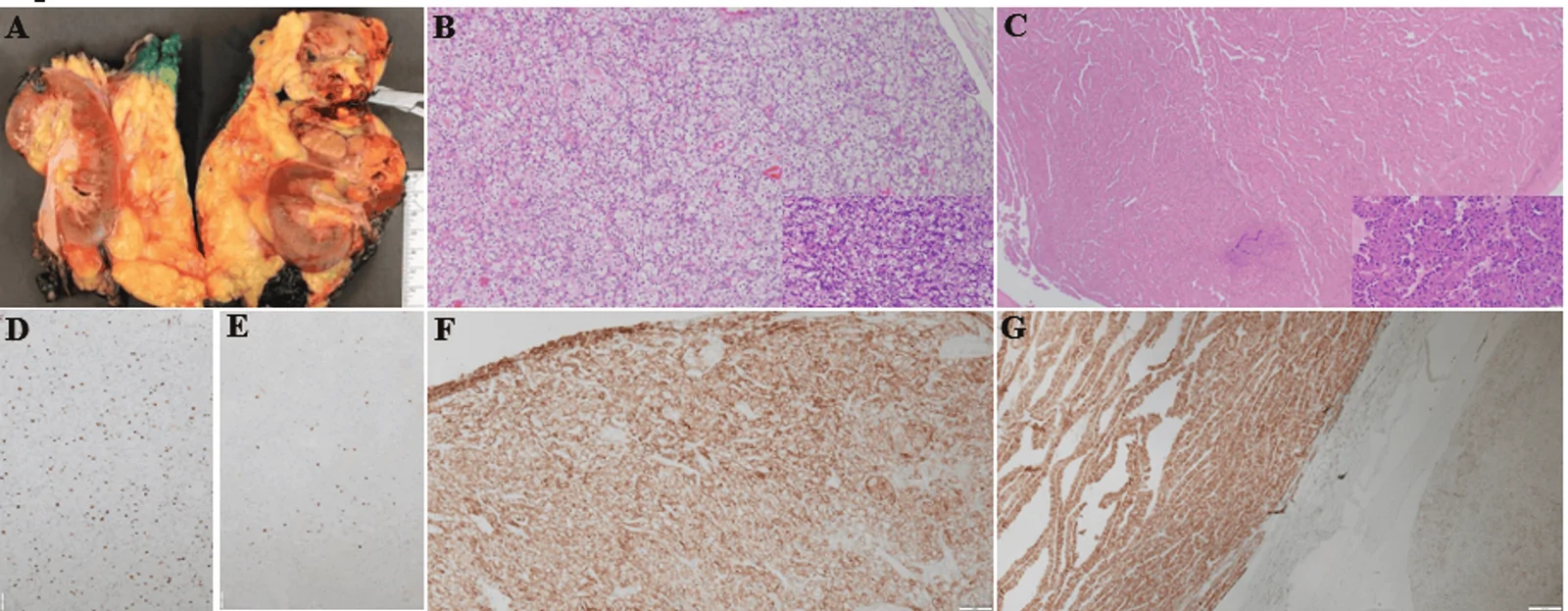
Morphologic and immunohistochemical features of the collision tumor composed of clear cell renal cell carcinoma (ccRCC) and papillary renal cell carcinoma (pRCC). (A) Gross photograph of the bisected left nephrectomy specimen demonstrating two well-demarcated, spatially adjacent tumors in the upper pole: a golden-yellow tumor with focal hemorrhage consistent with ccRCC (3.3 cm) and a homogeneous tan-gray tumor consistent with pRCC (3.2 cm). (B) Representative histologic appearance of the ccRCC component with an inset showing higher magnification (H&E stain). (C) Representative histologic appearance of the pRCC component with an inset showing higher magnification (H&E stain). (D) Ki-67 immunohistochemistry demonstrating a higher proliferative index in the ccRCC component. (E) Ki-67 immunohistochemistry of the pRCC component showing a lower proliferative index. (F) CAIX immunohistochemistry showing diffuse membranous positivity in the ccRCC component. (G) AMACR immunohistochemistry demonstrating diffuse, strong cytoplasmic granular positivity in the pRCC component (left) and only patchy weak staining in the ccRCC component (right).

The left radical nephrectomy specimen weighed 434.5 g and measured 14.5 x 10.4 x 6.5 cm. Gross examination revealed two well-demarcated, spatially adjacent tumors at the upper pole. The tumor with a golden-yellow cut surface and focal hemorrhage (ccRCC) measured 3.3 x 2.1 x 2.0 cm; the second one with a homogeneous tan-gray cut surface (pRCC) measured 3.2 x 2.1 x 1.5 cm (Figure 1A). The 6.3 cm dimension reported on preoperative CT reflects the combined radiographic impression of the two contiguous tumors, which were not resolved as separate lesions on imaging; the 3.3 cm and 3.2 cm measurements above were obtained individually at gross examination, once the specimen was bisected and the two components could be distinguished by their differing cut surfaces.

Microscopic examination confirmed two histologically distinct tumors characteristic of clear cell and papillary morphology, respectively (Figure 1B-1C). The only salient differential immunohistochemical markers are CAIX, which is diffusely positive for ccRCC (WHO/ISUP Grade 2) and completely negative for pRCC; and AMACR, where pRCC showed diffuse cytoplasmic granular staining while ccRCC had only patchy weak cytoplasmic positivity, as shown in Figure 1F-1G. Other markers such as CK7, CD10, PAX8 and vimentin played no role in differentiating the two tumors. CD117 negativity ruled out chromophobe RCC. No lymphovascular invasion was identified in either of the tumors.

To identify the genetic features of the collision tumor, separate NGS profiling was performed on each component using the Tempus xT 648-gene DNA panel and xR RNA platform. No matched normal tissue was available. Results are summarized in Table 1. The pRCC harbored RHEB p.Y35C (VAF 34.7%) and TERT c.-124C>T (VAF 30.3%) with TMB-High status (10.4 mut/Mb). The ccRCC contained PBRM1 p.C1232Vfs*12 (VAF 21.3%), TGFBR1 p.E2* (VAF 22.5%), and ARID1A p.P2075Hfs*60 (VAF 15.5%) with TMB 9.2 mut/Mb. Both were microsatellite stable. No shared somatic mutations or RNA fusions were identified.

**Table 1:** Next-generation sequencing findings for each tumor component obtained from separate profiling of the papillary RCC and clear cell RCC components. VAF: variant allele fraction; TMB: tumor mutational burden; MSI: microsatellite instability; IPS: immune profile score.

Feature	Papillary RCC	Clear Cell RCC
Key mutations	RHEB p.Y35C (VAF 34.7%); TERT c.-124C>T (VAF 30.3%)	PBRM1 p.C1232Vfs*12 (VAF 21.3%); TGFBR1 p.E2 (VAF 22.5%); ARID1A p.P2075Hfs*60 (VAF 15.5%)
Tumor mutational burden (TMB)	10.4 mutations/Mb (TMB-High)	9.2 mutations/Mb (TMB-Low)
Microsatellite instability (MSI) status	Microsatellite stable	Microsatellite stable
Immune profile score (IPS)	48 (IPS-High)	Not separately reported
RNA sequencing	No rearrangements or aberrant splicing events detected	No rearrangements or aberrant splicing events detected
Biomarker-informed therapeutic implications	Pembrolizumab (TMB-High tissue-agnostic indication)	Nivolumab sensitivity potentially associated with PBRM1 loss-of-function alterations in ccRCC
Predominant molecular drivers	mTOR pathway activation (RHEB); telomerase reactivation (TERT)	SWI/SNF chromatin-remodeling deficiency (PBRM1, ARID1A); TGF-β signaling disruption (TGFBR1)
Shared mutations	None identified	None identified

## Discussion

The completely non-overlapping somatic mutation profiles confirm that the ccRCC and pRCC arose through independent oncogenic events, establishing this as a true collision tumor. The principal differential diagnosis includes composite (mixed-subtype) RCC and clear cell papillary renal cell tumor (CCPRCT) [7,11]. Composite tumors share the exact same primary driver mutations and genetic alterations despite divergent histology; CCPRCT is characterized by diffuse CK7 and CAIX positivity with negative CD10 and AMACR staining [12,13]. The papillary RCC in the present case, with diffuse AMACR expression and being negative for CK7 and CAIX, is inconsistent with CCPRCT. Despite identical staging and negative margins, the ccRCC component of the collision tumor exhibited a higher proliferation index by Ki-67 than the adjacent pRCC (Figure 1D-1E). The higher Ki-67 index in the ccRCC component may suggest greater proliferative activity, though the prognostic significance of this finding in the context of collision tumors is uncertain.

The largest series of renal collision tumors reported that ccRCC-pRCC is among the most common malignant combinations, though NGS-based characterization was not performed in most cases [6]. The present case demonstrates the distinct molecular characteristics of a collision ccRCC-pRCC via comprehensive, component-specific sequencing, an approach documented in only a limited number of published reports [14].

Molecular testing does not always confirm a morphologic impression of collision. In one reported case, a renal tumor with mixed clear cell, eosinophilic, and papillary morphology - initially suspected on histology to represent a collision tumor - was reclassified by whole-exome sequencing as a single, unifying TFEB/6p21/VEGFA-amplified entity rather than independently colliding neoplasms [15]. By contrast, component-specific sequencing in the present case demonstrated completely non-overlapping somatic alterations between the ccRCC and pRCC components, providing direct molecular evidence of independent clonal origins rather than a single entity with divergent morphology.

The pRCC component was found to harbor an oncogenic, gain-of-function RHEB Y35C mutation, resulting in a tyrosine-to-cysteine substitution at position 35. This hyperactivating alteration renders the RHEB protein (a small GTPase) resistant to GTP hydrolysis, leading to constitutive mTORC1 activation. RHEB mutations at position 35 drive downstream mTORC1 signaling and, in specific variants, promote MEK-ERK pathway co-activation via altered BRAF interactions. Consequently, the RHEB Y35C variant acts as a dual-pathway driver, unleashing aberrant MEK-ERK signaling to accelerate cell proliferation [14]. Concurrently, the TERT promoter mutation upregulates telomerase expression, maintaining telomere length and conferring replicative immortality on proliferating tumor cells and resistance to replicative senescence [16-18]. In contrast, the ccRCC component demonstrated loss-of-function alterations in three established genomic drivers of clear cell renal cell carcinoma pathogenesis. PBRM1, encoding the BAF180 subunit of the PBAF chromatin-remodeling complex, is mutated in approximately 40% of ccRCC cases. Its inactivation disrupts regulation of the hypoxia-inducible factor (HIF) signaling cascade, driving aberrant metabolic reprogramming and unchecked proliferation. ARID1A, another essential SWI/SNF complex subunit, cooperates with PBRM1 in maintaining chromatin accessibility at genomic enhancers; its loss impairs transcriptional regulation and DNA damage repair while promoting cell migration and tumor progression. The truncating TGFBR1 alteration abrogates TGF-β receptor signaling, rendering tumor cells refractory to physiological growth-inhibitory signals and further enabling the autonomous proliferative phenotype characteristic of ccRCC [11]. Collectively, these alterations converge on chromatin dysregulation and loss of growth restraint, a molecular signature distinct from the mTOR/TERT-driven oncogenesis observed in the pRCC component.

The distinct molecular profiles in this case highlight important, component-specific therapeutic implications. The pRCC component met the clinical criteria for pembrolizumab eligibility based on a high tumor mutational burden (TMB-High) status. Conversely, the ccRCC component harbored a loss-of-function PBRM1 alteration; although still debated, this biomarker has been associated with enhanced clinical benefit from immune checkpoint blockade in select ccRCC cohorts [10,19,20]. It should be noted that these biomarker findings did not influence actual management in this patient, who was treated with surgery alone and did not receive systemic therapy; their relevance here is illustrative of what component-specific profiling could inform in future cases rather than a demonstrated clinical effect in this one.

Limitations

This report has several limitations. As a single case, our findings are not generalizable, and the therapeutic implications discussed above should be considered hypothesis-generating rather than clinically validated in this patient. Sequencing was performed on tumor tissue alone, without matched normal tissue for germline subtraction; this tumor-only sequencing approach means that a contribution from germline variants or clonal hematopoiesis to the lower-variant-allele-fraction alterations cannot be entirely excluded, and the identified alterations should be interpreted as somatic-enriched rather than confirmed-somatic findings. Long-term oncologic follow-up beyond the surveillance period reported here is not yet available.

## Conclusions

Synchronous, histologically distinct clear cell and papillary renal cell carcinomas within a single kidney can represent true collision tumors with independent clonal origins rather than a single, divergent tumor. Component-specific next-generation sequencing distinguished separate molecular profiles in this case, revealing distinct oncogenic drivers, including RHEB and TERT mutations in the papillary component and PBRM1/ARID1A/TGFBR1 alterations in the clear cell component. These findings suggest that separate, biomarker-driven therapeutic approaches may be warranted in similar cases, though this remains to be validated in larger, prospective cohorts.
